# Analysis of immunophenotypic features in hyaline vascular type Castleman disease

**DOI:** 10.1186/s13000-023-01421-w

**Published:** 2023-12-07

**Authors:** Yu Chang, Yu Ma, Chen Chang, Wensheng Li

**Affiliations:** 1 Department of Pathology, Shaanxi Provincial People’s Hospital, The Third Affiliated Hospital of Xi’an Jiaotong University Health Science Center, Xi’an, 710068 China; 2 Institute of Medical Research, Northwestern Polytechnical University, Xi’an, 710068 China

**Keywords:** Hyaline vascular type Castleman Disease, Immunophenotypic features, CD3, CD21, BCL2

## Abstract

**Background:**

Studies suggested that the immunophenotype of hyaline vascular type Castleman disease (HVCD) lacked characteristics, which was easy to be confused with other diseases.

**Methods:**

From January 2010 to June 2022, 17 cases of HVCD were selected from the Department of Pathology of Shaanxi Provincial People’s Hospital and the Department of Pathology of Shaanxi Provincial Cancer Hospital. 13 cases of reactive hyperplastic lymph nodes (RHL) and 11 cases of follicular lymphoma (FL) were selected as the control group. All cases were performed CD3, CD20, CD21 and BCL2 immunohistochemical staining.

**Results:**

(i) In 17 cases of HVCD, the negative area of BCL2 of germinal center was significantly smaller than the negative area of CD3 of germinal center. However, in 13 cases of RHL, the negative area of CD3 of germinal center was basically consistent with the negative area of germinal center of BCL2 of germinal center. In 11 cases of FL, in neoplastic follicles, the negative area of CD3 was basically consistent with the positive area of BCL2. The difference between HVCD group and other two groups of diseases was statistically significant (*P* < 0.05). (ii) In 17 cases of HVCD, the negative area of BCL2 of germinal center was significantly smaller than the follicular dendritic cell (FDC) meshworks expressed by CD21. However, in 13 cases of RHL, the FDC meshworks expressed by CD21 were basically consistent with the negative area of BCL2 of germinal center. In 11 cases of FL, in neoplastic follicles, the FDC meshworks expressed by CD21 was basically consistent with the positive area of BCL2. The difference between HVCD group and other two groups of disease was statistically significant (*P* < 0.05).

**Conclusions:**

HVCD has unique immunophenotypic characteristics. The negative area of BCL2 of germinal center is significantly smaller than the negative area of CD3. The negative area of the BCL2 of germinal center is significantly smaller than the FDC meshworks expressed by CD21. These two immunophenotypic features in HVCD are very important in diagnosis and differential diagnosis.

## Introduction

Castleman disease (CD) is a group of rare and chronic lymphoproliferative disorders presenting as enlarged lymph nodes with clinical manifestation [1]. Lymph node biopsy is the reference standard for diagnosing CD [2]. Histopathological classifications of CD range from what is considered to be hyaline vascular (HV) type to plasmacytic (PC) type, with a mixed type in between that exhibits both features. HVCD is the most common histological subtype, which is easy to be confused with reactive hyperplastic lymph nodes (RHL) and follicular lymphoma (FL).

The immunophenotypes of HVCD reported were CD3 and CD20 showed normal distribution of T and B cells. CD3 immunohistochemical staining was positive in the interfollicular region. CD20 was positive in the lymphoid follicles. CD21 and CD23 were positive in FDC meshworks [3, 4]. Previous studies on HVCD immunophenotype were limited to whether T and B cells were expressed in the normal part of the lymph node, whether FDC meshworks existed, and whether the lymph node structure was destroyed. Studies suggested that the immunophenotype of HVCD lacked characteristics. Therefore, this study aims to further study the immunophenotype of HVCD, and attempt to find the immunophenotype characteristics of HVCD, providing a new idea for the diagnosis and differential diagnosis of HVCD.

## Materials and methods

### Case selection

From January 2010 to June 2022, 17 cases of HVCD were selected from the Department of Pathology of Shaanxi Provincial People’s Hospital and the Department of Pathology of Shaanxi Provincial Cancer Hospital. 13 cases of reactive hyperplastic lymph nodes (RHL) and 11 cases of follicular lymphoma (FL) were selected as the control group. All sections were reevaluated by two senior pathological attending physicians.

### Methods

All specimens were fixed with 10% neutral formalin solution, dehydrated, embedded in paraffin wax, and sliced with a thickness of 4 μm. Hematoxylin and eosin (HE) staining and immunohistochemical staining were performed. All primary antibodies including CD3 (Clone MX036), CD20 (Clone L26), CD21 (Clone MX019) and BCL2 (Clone MX022) and second antibody (mouse-anti-human antibody) were ready-to-use and purchased from Fuzhou Maixin Biotechnology Development Co., LTD, Fuzhou, China. Sections were stained using MaxVision method. Antigen retrieval was performed by pressure-cooking the slides in EDTA/Tris-HCl buffer or citrate buffer for one and a half min. Sections were incubated for 60 min each with the primary antibody, and 15 min with the second antibody. DAB staining was done for 5 min. All reactions were carried out at 37℃. Tonsil tissue was used for positive control of CD3, CD20, CD21 and BCL2. PBS was used for negative control.

### Interpretation of immunohistochemical results

The staining sites of CD3, CD20 and CD21 were in cell membrane, which of BCL2 were in cytoplasm or cell membrane. T cells by CD3 stain were mainly distributed in the interfollicular region, and a small number of positive T cells were also seen in the germinal center. CD20 was expressed in B cells, positive for lymphoid follicles and interfollicular zone. CD21 was positive in FDC meshworks, and also weakly expressed by mantle cells. BCL2 showed the germinal center was negative. Sections positive in the normal part were judged as positive in five different HPFs.

### Statistical analysis

Statistical analyses using Fisher exact test were carried out by SPSS software v.25.0. A *P* value < 0.05 was considered statistically significant.

## Results

### Clinical features

In 17 cases of HVCD, the age ranged from 9 to 65 years with a mean age of 33 years. The ratio of male to female was 1.13:1, including 9 males and 8 females. Cervical lymph nodes were the most common sites of involvement. 7 cases occurred in cervical lymph nodes, including 4 cases in left cervical lymph nodes, 3 cases in right cervical lymph nodes and 3 cases in mediastinal lymph nodes. Other cases occurred in inguinal lymph nodes, axillary lymph nodes, retroperitoneal lymph nodes, submaxillary lymph nodes, mesenteric lymph nodes, and left subclavian lymph nodes. The maximum diameter of the enlarged lymph nodes ranged from 0.9 to 5 cm with a mean value of 2.9 cm. Clinically, 13 cases of HVCD were a single enlarged lymph node, and 4 cases were systemic multiple enlarged lymph node.

### Histological features

**Fig. 1:**
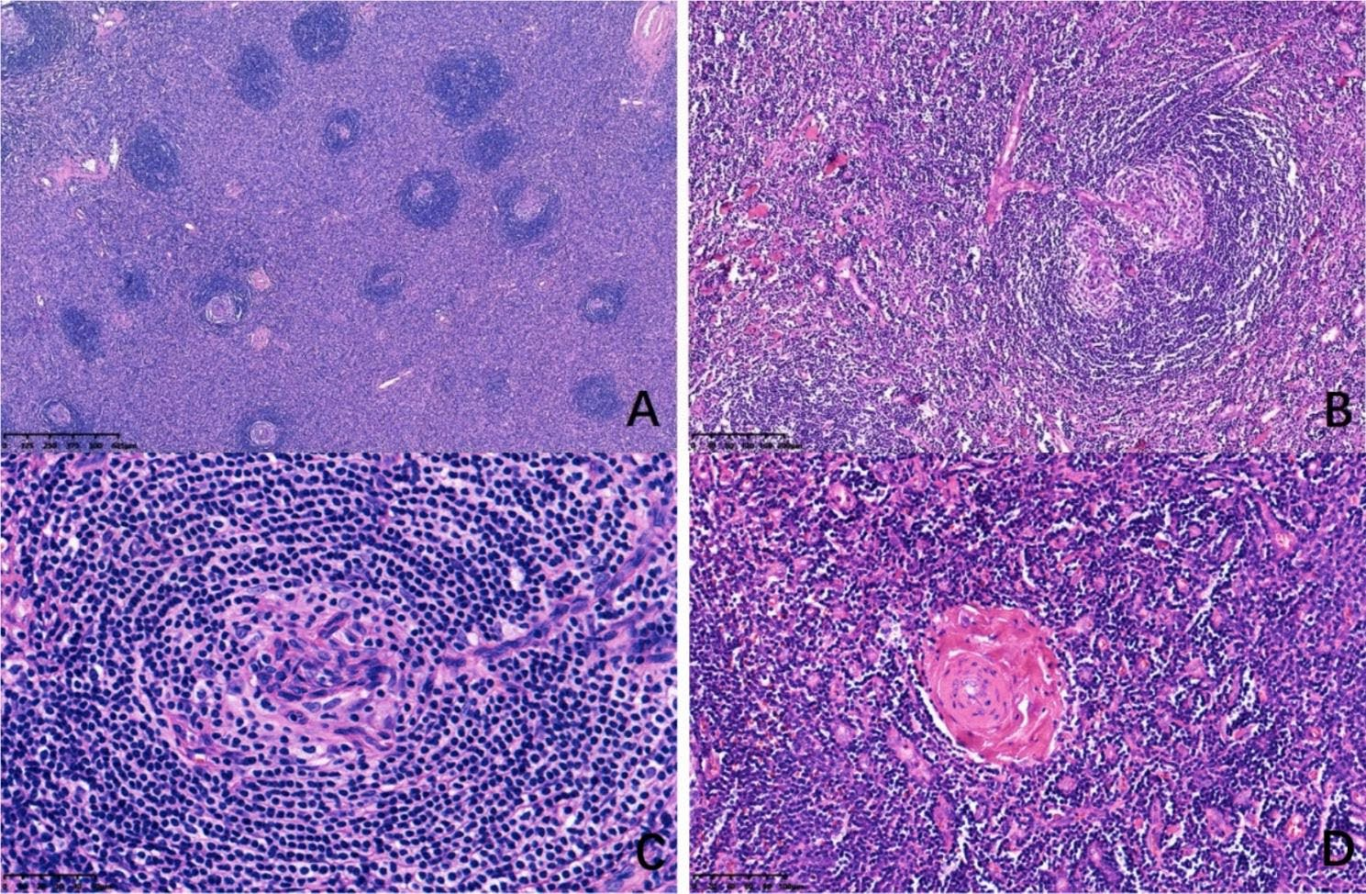
Histological features of HVCD (HE stain). **A**, Lymphoid follicles are relatively uniform in size, with the depleted germinal center. **B**, multiple germinal centers. **C**, onion skin appearance and lollipop lesions. **D**, increased small vessels in the interfollicular region

The histological characteristics of 17 HVCD cases were shown in Table 1. Microscopy showed increased lymphoid follicles in lymph nodes and depleted germinal center in follicles (Fig. 1A). Multiple germinal central follicles (Fig. 1B) and obvious widening of mantle zone were observed in 12 cases. 15 cases showed a concentric ring of lymphocytes with onion skin appearance (Fig. 1C). Small vessels in the interfollicular region of 17 cases were increased (Fig. 1D), and penetrated into the lymphoid follicles forming lollipop lesions (Fig. 1C). The lymphoid sinuses were significantly reduced or disappeared in 14 cases, but still present in 3 cases.

**Table 1:** Histological features in 17 cases of HVCD

Histological features	cases(%)
Follicular hyperplasia	17(100)
Depleted germinal centers	17(100)
Multiple germinal centers	12(71)
Onion skin appearance in mantle zone	15(88)
Lollipop lesions	17(100)
Reduced or disappeared lymphoid sinuses	14(82)

### Immunophenotypic characteristics of HVCD

The first unique immunophenotypic feature of HVCD: the negative area of BCL2 of germinal center was significantly smaller than the negative area of CD3.

**Fig. 2:**
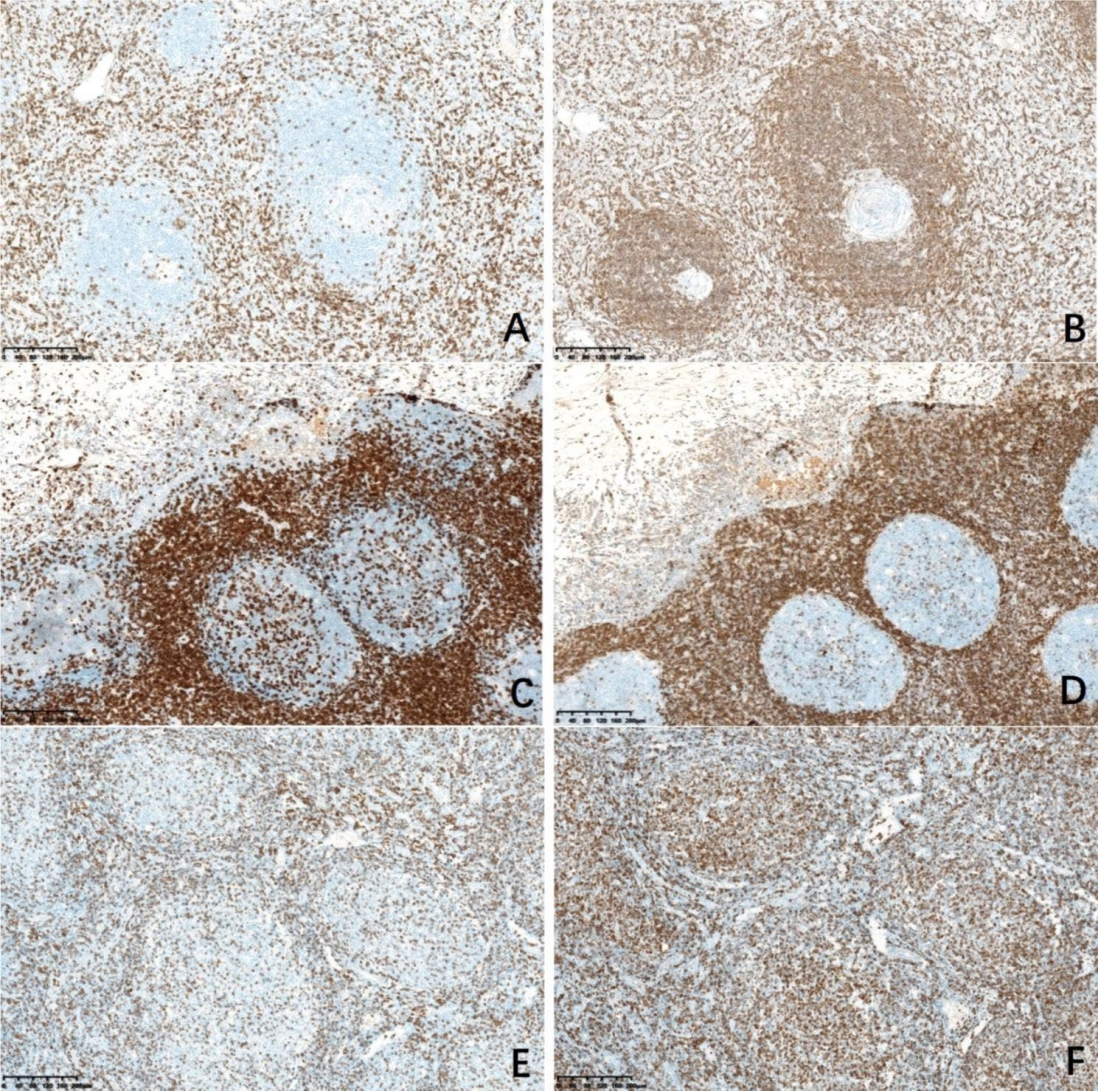
Immunohistochemical staining for CD3 and BCL2 in HVCD, RHL and FL. **A**, The lymphoid follicles were negative for CD3 in HVCD. **B**, BCL2 showed the germinal center was negative in HVCD. **C**, The lymphoid follicles were negative for CD3 in RHL. **D**, BCL2 showed the germinal center was negative in RHL. **E**, CD3 showed neoplastic B cells were negative in FL. **F**, BCL2 showed the neoplastic follicles were positive in FL

By comparing and observing the immunohistochemical staining of all cases of HVCD, RHL and FL, we found that the immunophenotype of HVCD has the following characteristics: In 17 cases of HVCD, the negative area of BCL2 of germinal center was significantly smaller than the negative area of CD3 (Fig. 2A, B). However, in 13 cases of RHL, the negative area of CD3 was basically consistent with the negative area of germinal center of BCL2 (Fig. 2C, D). In 11 cases of FL, in neoplastic follicles, the negative area of CD3 was basically consistent with the positive area of BCL2 (Fig. 2E, F). The difference of CD3 and BCL2 immunohistochemical staining expression area between HVCD and RHL groups was statistically significant (*P* < 0.05), and that between HVCD and FL groups was statistically significant (*P* < 0.05) (Table 2). The negative area of germinal center of BCL2 is significantly smaller than the negative area of CD3 in HVCD, which is the first unique immunophenotypic feature of HVCD.

**Table 2:** Comparison of CD3 and BCL2 immunohistochemical staining expression regions in HVCD, RHL and FL(cases, %)

Groups	Total(cases)	The CD3-negative area is clearly inconsistent with the BCL2-negative area	The CD3-negative area is basically consistent with the BCL2-negative area	The CD3-negative area is basically consistent with the BCL2-positive area	*P*
HVCD	17	17(100)	0(0)	0(0)	< 0.001
RHL	13	0(0)	13(100)	0(0)	
FL	11	0(0)	0(0)	11(100)	

*P* < 0.05 means the difference is statistically significant.

The second unique immunophenotypic feature of HVCD: the negative area of BCL2 of germinal center was significantly smaller than FDC meshworks expressed by CD21 stain.

**Fig. 3:**
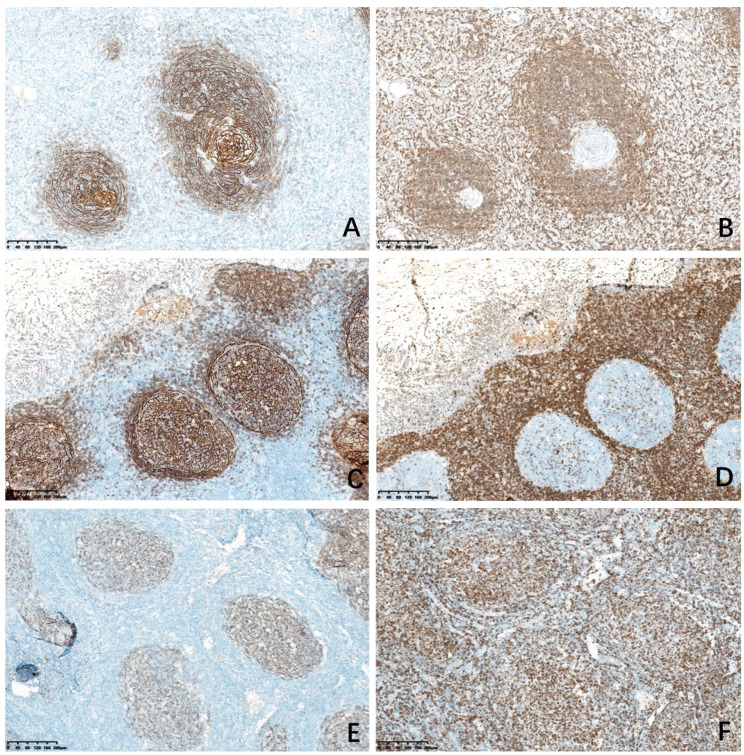
Immunohistochemical staining for CD21 and BCL2 in HVCD, RHL and FL. **A**, FDC meshworks by CD21 stain in HVCD. **B**, negative area of BCL2 of germinal center in HVCD. **C**, FDC meshworks by CD21 stain in RHL. **D**, negative area of BCL2 of germinal center in RHL. **E**, FDC networks of neoplastic follicles by CD21 stain in FL. **F**, positive neoplastic follicles of BCL2 in FL

By comparing and observing the immunohistochemical staining of all cases of HVCD, RHL and FL, we found that the immunophenotype of HVCD has the following characteristics: In 17 cases of HVCD, the negative area of BCL2 of germinal center was significantly smaller than the FDC meshworks expressed by CD21 stain (Fig. 3A, B). However, in 13 cases of RHL, the FDC meshworks expressed by CD21 stain were basically consistent with the negative area of BCL2 of germinal center (Fig. 3C, D). In 11 cases of FL, in neoplastic follicles, the FDC meshworks displayed by CD21 stain were basically consistent with the positive area of BCL2 (Fig. 3E, F). The difference between the CD21-positive area and the BCL2 negative area in HVCD and RHL groups was statistically significant (*P* < 0.05). The difference in HVCD and FL groups was also statistically significant (*P* < 0.05) (Table 3). The negative area of germinal center of BCL2 is significantly smaller than the FDC meshworks expressed by CD21 stain in HVCD, which is the second unique immunophenotypic feature of HVCD.

**Table 3:** Comparison of CD21 and BCL2 immunohistochemical staining expression regions in HVCD, RHL and FL(cases, %)

Groups	Total(cases)	The CD21-positive area is clearly inconsistent with the BCL2-negative area	The CD21-positive area is basically consistent with the BCL2-negative area	The CD21-positive area is basically consistent with the BCL2-positive area	*P*
HVCD	17	17(100)	0(0)	0(0)	< 0.001
RHL	13	0(0)	13(100)	0(0)	
FL	11	0(0)	0(0)	11(100)	

*P* < 0.05 means the difference is statistically significant

### Pathogenesis

We found that the negative area of germinal center of BCL2 was significantly smaller than the FDC meshworks expressed by CD21 stain in 17 cases of HVCD. The negative area of germinal center of BCL2 was reduced, but the FDC meshworks shown by CD21 was hyperplastic. It can be speculated that the formation mechanism of germinal center atrophy may be associated with the obvious widening of the mantle area. It is possible that the germinal center is reactive hyperplastic at the beginning. The mantle zone not only widens outwards, but also widens inwards and compresses the germinal center, leading to the atrophy of the germinal center.

## Discussion

Castleman disease is a group of rare and chronic lymphoproliferative disorders presenting as painless and enlarged lymph nodes with clinical manifestation, so it was named giant lymphadenopathy in the past. At present, the etiology and pathogenesis of CD are still unclear. In recent years, studies have shown that viral infections such as HHV-8, HIV, and EBV can cause activation of the autoimmune system, resulting in the release of cytokines such as IL-6, and hyperproliferation of B lymphocytes and plasma cells in lymphoid tissues.

The pathological types of CD include HVCD, PCCD and mixed CD. Among them, HVCD is the most common pathological type. The histological features are mainly atrophy of the germinal center in follicles, and onion skin appearance in mantle zone. Hyaline vessels penetrate the follicles to form lollipop lesions. Increased numbers of small vessels in the interfollicular area are seen, and the lymphatic sinuses disappear.

The histological features of PCCD are lymphoid follicular hyperplasia, and a large number of mature plasma cells can be seen in the interfollicular area. While the proliferation of small vessels is not obvious, and there is no typical onion skin appearance. Mixed CD has the histological features of both HVCD and PCCD.

CD has not been found to have specific immune markers and is lack of immunophenotypic characteristics. Immunohistochemical staining shows CD20 is positive in follicles. CD3 is positive in interfollicular area. BCL2 is negative in germinal center. CD21, CD35 and CD23 show the FDC meshworks.

Histopathological examination of enlarged lymph nodes is regarded as the glad standard to diagnose CD. When diagnosing CD, lymph nodes with CD-like lesions must be excluded [5], including infectious diseases, autoimmune diseases, and malignancies, such as RHL and FL.

RHL is mainly manifested as lymph nodes enlargement, which can be divided into two types: one is specific RHL caused by infections such as Mycobacterium tuberculosis or fungi, and the other is simple RHL, including follicular hyperplasia type, paracortical hyperplasia type and sinus histiocytosis type. Microscopic examination shows the lymph nodes have normal structures and hyperplastic follicles varied in size. Hyperplastic germinal centers are polarized, displaying a centroblast-rich dark zone with brisk mitotic activity and tingible-body macrophages [6]. Mitotic figures are easy to see. There is a thin lymph node capsule and lymphatic sinuses. The RHL immunophenotype shows the normal distribution of T and B lymphocytes in their certain distribution area [7].

FL is a non-Hodgkin lymphoma with t(14;18)(q32;q21) translocation and *BCL2* mutation, and FL originates from germinal center B-cells [8]. Microscopically, partial or complete effacement of the lymph node architecture with numerous and dissimilarly sized neoplastic follicles. Neoplastic follicles with attenuated or absent mantle zones are back-to-back and lack nonpolarized. There are centrocytes and larger centroblasts in neoplastic follicles, in which are few or absent tingible body macrophages. Contraction and distortion of FDC meshworks have also been described [9]. The centrocytes are small to medium in size with scant cytoplasm and inconspicuous nucleoli. The centroblasts are large in size with round or oval nuclei, vesicular chromatin and 1–3 peripheral nucleoli [10]. Normally, neoplastic follicles express CD20 and BCL2, whereas there are partly positive or negative for BCL2 in approximately 15 -25% of grade 2 and 3 FL cases [11].

In general, HVCD can be distinguished from RHL and FL combining the clinical manifestations and typical pathological features. However, RHL and FL sometimes show histopathological features similar to HVCD, causing misdiagnosis [12, 13]. Follicular subtype of HVCD shows hyperplastic follicles are more and denser than other types and account for more than 50% of the lymph node, which is reminiscent of FL at low magnification [14]. When the germinal centers of lymphoid follicles in HVCD are obviously shrunk to disappear or cannot be displayed due to section reasons, the follicles are positive for BCL2, which is also easy to be misdiagnosed as FL. Unusual morphological variants of FL can sometimes show histopathological features reminiscent of HVCD, such as regressive germinal centers, onion skin of broad mantle zone and hyaline vessels inserted into follicles [15, 16]. Thus, it is a challenge to differentiate HVCD from RHL and FL.

Therefore, this study conducted further observation and research on the immunophenotype of HVCD, and found for the first time that HVCD has two unique immunophenotypic characteristics: The negative area of germinal center of BCL2 is not only significantly smaller than the negative area of CD3, but also significantly smaller than the FDC meshworks expressed by CD21. These two immunophenotypic features of HVCD can be used for differential diagnosis with RHL and FL.

Immunohistochemically, the four regions of CD3-negative region, CD20-positive follicular region, CD21-positive region showing FDC meshworks, and BCL2 germinal center negative region are basically similar, which suggests the diagnosis of RHL. When the BCL2 germinal center negative area is not only significantly smaller than the CD3 negative area, but also significantly smaller than the CD21-marked FDC meshworks, which suggests the diagnosis of HVCD. In addition, these two immunophenotypic characteristics of HVCD can also be differentiated from FL. The immunophenotype of FL is that CD3-negative region and CD21-positive region showing FDC meshworks are basically consistent with the BCL2-positive region, which is obviously different from HVCD. Therefore, we think that the unique immunophenotypic characteristics of HVCD can be used as an important basis for the differential diagnosis of RHL and FL.

Otherwise, data suggests that unicentric CD is more likely a clonal neoplastic process, and the most likely cell of origin is stromal, specifically the FDC [17]. In this study, the negative area of BCL2 of germinal center is reduced, which is significantly smaller than the CD21-expressed FDC meshworks. We speculate that the mantle zone not only widens outwards, but also widens inwards and compresses the germinal center, which may lead to the atrophy of the germinal center. It is helpful to improve the understanding of CD, and suggests the possible mechanism of CD.

In conclusion, this study finds HVCD has two unique immunophenotypic features: the negative area of germinal center of BCL2 is significantly reduced, which is not only significantly smaller than the CD3-negative area, but also significantly smaller than the CD21-expressed FDC meshworks. The two unique immunophenotypic features in HVCD have a key role in diagnosis and differential diagnosis.

## Abbreviations

CD: Castleman disease
FDC: Follicle dendritic cell
FL: Follicular lymphoma
HVCD: Hyaline vascular type Castleman disease
PCCD: Plasma cell type Castleman disease
RHL: Reactive hyperplastic lymph nodes

## References

[1] Dispenzieri A, Fajgenbaum DC (2020). Overview of Castleman Disease. Blood.

[2] Gündüz E, Özdemir N (2021). A rare lymphoproliferative Disease: Castleman Disease. Turk J Haematol.

[3] Yunzhu L (2019). Primary hyaline vascular Castleman Disease of the kidney: case report and literature review. Diagn Pathol.

[4] Sevilla-Lizcano DB, Frias-Soria CL, Ortiz-Hidalgo C (2017). Castleman Disease. Histopathological and immunohistochemical analysis of 39 cases. Gac Med Mex.

[5] Wu DLM, Jaffe ES (2018). Pathology of Castleman Disease. Hematol Oncol Clin North Am.

[6] Tzankov A, Dirnhofer S (2018). A pattern-based approach to reactive lymphadenopathies. Semin Diagn Pathol.

[7] Gars E, Butzmann A, Ohgami R, Balakrishna JP, O’Malley DP (2020). The life and death of the germinal center. Ann Diagn Pathol.

[8] Khanlari M, Chapman JR (2022). Follicular Lymphoma: updates for pathologists. J Pathol Transl Med.

[9] Nann D, Ramis-Zaldivar JE, Müller I (2020). Follicular Lymphoma t(14;18)-negative is genetically a heterogeneous Disease. Blood Adv.

[10] Randall C, Fedoriw Y (2020). Pathology and diagnosis of follicular Lymphoma and related entities. Pathology.

[11] Marafioti T, Copie-Bergman C, Calaminici M (2013). Another look at follicular Lymphoma: immunophenotypic and molecular analyses identify distinct follicular Lymphoma subgroups. Histopathology.

[12] van Rhee F, Oksenhendler E, Srkalovic G (2020). International evidence-based consensus diagnostic and treatment guidelines for unicentric Castleman Disease. Blood Adv.

[13] Post GR, Bell RC, Rjoop A, Lobo RH, Yuan Y, Post SR (2016). Diagnostic utility of Interleukin-6 expression by immunohistochemistry in differentiating Castleman Disease subtypes and reactive lymphadenopathies. Ann Clin Lab Sci.

[14] Kojima M, Shimizu K, Ikota H (2008). Follicular variant of hyaline-vascular type of Castleman’s Disease: histopathological and immunohistochemical study of 11 cases. J Clin Exp Hematop.

[15] Aladily TN, Al-Fararjeh F, Bustami N, Mansour AT (2022). Follicular Lymphoma behind a facade of Castleman Disease, a diagnostic challenge. Indian J Pathol Microbiol.

[16] Jiwon K, Kyung JY (2020). Morphologic variant of follicular Lymphoma reminiscent of hyaline-vascular Castleman Disease. J Pathol Translational Med.

[17] Chang KC, Wang YC, Hung LY (2014). Monoclonality and cytogenetic abnormalities in hyaline vascular Castleman Disease. Mod Pathology: Official J United States Can Acad Pathol Inc.

